# Beyond the surface: Distinguishing leprosy from fungal mimics - A case report

**DOI:** 10.51866/cr.954

**Published:** 2025-11-17

**Authors:** Nurjasmine Aida Jamani, Norrifhan Akmal Ismail, Mohd Shaari Nur Shairah

**Affiliations:** 1 MBBS, Family Health Clinic IIUM, Department of Family Medicine, Kuliyyah of Medicine, International Islamic University of Malaysia, Jalan Sultan Ahmad Shah, Bandar Indera Mahkota, Kuantan, Pahang, Malaysia. E-mail: norrifhan91@gmail.com; 2 MD, MMED (FamMed), Department of Family Medicine, Kuliyyah of Medicine, International Islamic University of Malaysia, Jalan Sultan Ahmad Shah, Bandar Indera Mahkota, Kuantan, Pahang, Malaysia.; 3 MBBCh, MMED (FamMed), Klinik Kesihatan Tersang, Felda Tersang, Raub, Pahang, Malaysia.

**Keywords:** Hypopigmentation, Leprosy, Misdiagnosis

## Abstract

Leprosy, a chronic infectious disease caused by *Mycobacterium leprae,* remains a significant public health concern in many developing countries, including Malaysia. Continuous monitoring and effective diagnostic strategies are crucial for curbing its transmission. The insidious nature of leprosy, characterised by slow progression and diverse clinical manifestations, often leads to delayed diagnosis, increasing the risk of irreversible nerve damage and subsequent disabilities. We report the case of a 55-year-old man who had leprosy presenting with chronic generalised hypopigmented lesions and was misdiagnosed with a fungal infection. Early detection and accurate differentiation from other hypopigmented skin conditions are crucial for timely intervention and the prevention of long-term complications.

## Introduction

Chronic skin lesions are a common presentation in primary care practice. Leprosy, also known as Hansen’s disease is a chronic infectious disease caused by *Mycobacterium leprae* and *M. lepromatosis.* Although now rare, it remains endemic in some areas including Malaysia. This disease primarily affects the skin, peripheral nerves, mucosa of the upper respiratory tract and eyes, potentially leading to significant disability and deformities if left untreated.^1^

Leprosy remains a public health concern, particularly in low- and middle-income countries. Malaysia officially eliminated leprosy as a public health issue in 1994. Still, new cases continue to emerge, with 142 reported in 2021, 183 in 2022 and 256 in 2023.^2^ This makes leprosy a re-emerging disease of concern, warranting continuous surveillance, early detection and sustained public health efforts to prevent transmission and reduce the disease burden.^2^ In response, the World Health Organization (WHO) launched the ‘Towards Zero Leprosy’ initiative to eliminate the disease globally by 2030.^3^

Leprosy affects individuals across all age groups and is believed to spread through prolonged respiratory contact, though its transmission mechanisms remain incompletely understood. Leprosy is curable, and early treatment can prevent disability.^1^ Clinical manifestations may appear within a year or after several decades, typically involving chronic skin lesions and peripheral nerve involvement.^1^ Diagnosis is frequently delayed due to its resemblance to other conditions and limited awareness in nonendemic regions.^4,5^ We present the case of a man whose initial presentation of chest discomfort led to the incidental finding of leprosy. This case highlights the importance of considering leprosy in the differential diagnosis of chronic hypopigmented skin lesions, especially in endemic areas.

## Case presentation

**Figure 1:**
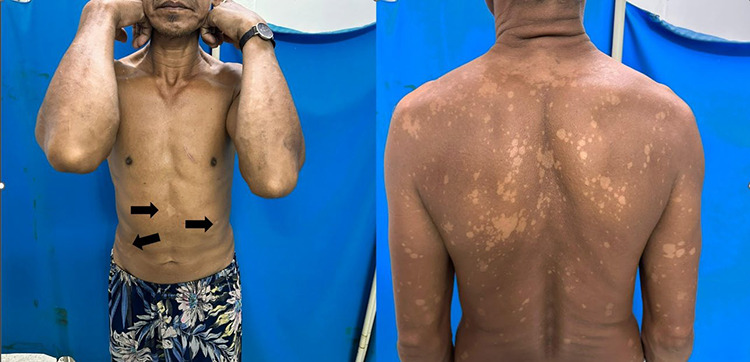
(Left) Clinical photograph demonstrating hypopigmentation over the abdomen and groin area (shown with arrows), with no visible thickened nerve seen over the bilateral elbows. (Right) Generalised hypopigmented skin lesions are concentrated more over the trunk region and bilateral upper limbs.

A 55-year-old man presented with a 2-day history of pleuritic chest discomfort. Clinical evaluation confirmed a diagnosis of costochondritis. On examination, multiple hypopigmented macules were incidentally noted on the hands, back, and trunk (Figure 1), reportedly present for five years. Despite multiple healthcare visits and empirical antifungal treatment, the lesions persisted without improvement. The lesions, initially confined to the trunk, gradually became more widespread and pruritic, necessitating further dermatological assessment.

Further history revealed no domestic or international travel, no contact with immigrants from endemic regions, or known exposure to leprosy cases or armadillos. He lives in a terrace house with his wife, son and two grandchildren, with good sanitation and no evidence of overcrowding. He was working as a farmer in a rural area.

**Figure 2:**
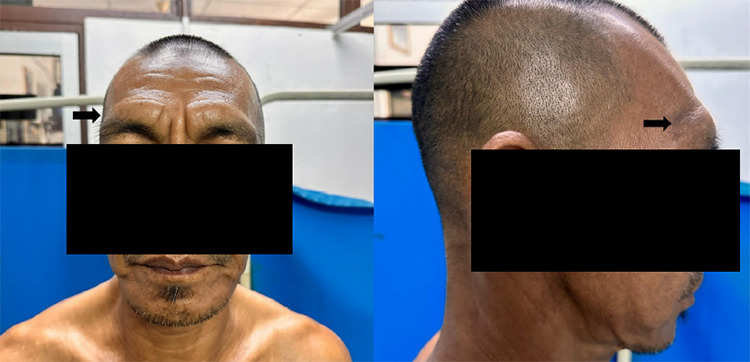
Clinical photographs showing thickened skin over the forehead and eyebrows, characteristic til^7^ leonine facies in lepromatous leprosy.

Physical examination revealed normal vital signs. He exhibited thickened forehead skin folds with preserved eyebrows (Figure 2). Multiple hypopigmented macules were symmetrically distributed over the upper trunk and back, with some coalescing into larger patches. The lesions were neither thickened nor associated with palpable nerves, and sensory testing showed no significant loss over the affected areas. There was no evidence of peripheral neuropathy, digital ulcers, or resorption. Other systemic examinations were unremarkable.

Slit-skin smears were obtained from both earlobes and the left shoulder to exclude leprosy, with tinea versicolor as *a* fifferential diagnosis. Acid-fast staining demonstrated acid-fast bacilli in all specimens, confirming multibacillary leprosy.

**Figure 3:**
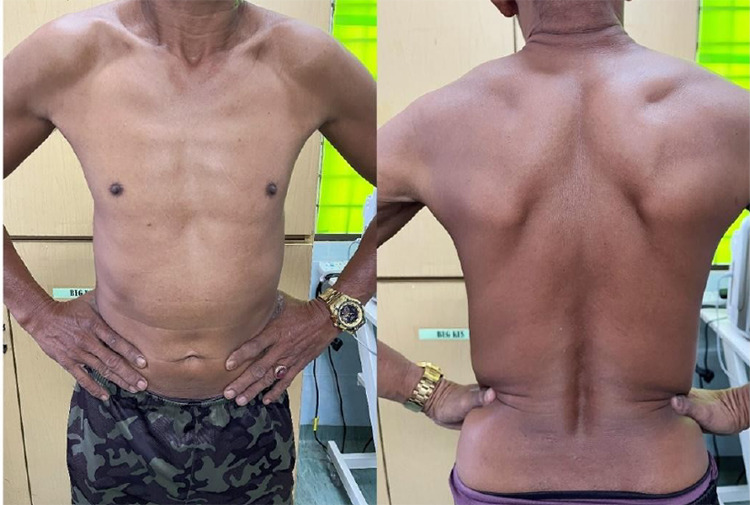
Clinical photograph demonstrating complete clearance of the hypopigmented lesions after 6 months of multidrug treatment for leprosy.

He was then treated with oral rifampicin 600 mg monthly, oral clofazimine 300 mg monthly and 50 mg daily and oral dapsone 100 mg daily. Before starting treatment, he was counselled on potential side effects, including the rare rilk of ftevens-Johnson syndrome, and was advised to seek mmediate care. On follow-up, he showed significant improvement in his skin lesions without neurological deficits (Figure 3). He was counselled on the importance of completing the full 12-month course of therapy and the need for regular monitoring for signs of relapse or disability. Additionally, contact tracing and screening of family members showed no evidence of leprosy.

## Discussion

Leprosy often mimics various dermatological and neurological conditions, making diagnosis challenging, especially for less experienced healthcare ‘workers.^4^ Common misdiagnoses include tinea versicolor, pityriasis alba, vitiligo, post-inflammatory hypopigmentation (PIH) and mycosis fUngoides (MF).^6^ As in this case, the patient visited multiple clinics over 5 years before being; correctly diagnosed. In 2023, Chen et al. found that approximately 44% of leprosy cases in their study were initially misdiagnosed, with the longest delay in accurate diagnosis reaching up to 67 months.^7^ Another study showed that leprosy ‘was often; misdiagnosed due to its ‘wide spectrum of appearance that often mimicked many other dermatological conditions.^6^

The three eardinal signs for the clinical diagnosis ofleprosy include anaesthetir or hypoanaesthetic skin lesions, thickened peripheral nerves with sensory impairment and detection of AFB in the skin smears.^5^ However, not all patients exhibit these classical signs, leading to delayed recognition, as occurred in our case. The manifestations of leprosy are highly variable based on the host’s immune response to *M. leprae*.^8^

The clinical spectrum reflects the host’s immune response to *Mycobacterium leprae.* Strong cell-mediated immunity results in teberculoid leprosy, characterired by few ‘well-defined hypopigmented patches, sensory loss, and minimal bacterial load. In contrast, weak immunity leads to lepromatous leprosy (LL), presenting with numerous symmetrical lesions, nodules, nerve involvement, and high bacterial burden. Borlerline Worms exhibit mixed features and may shift toward eitherpole depending on immune fluctuations. Indeterminate leprosy, an early form, manifests as vague hypopigmented parches and may progress based on immune response.^8^ In this case, the presence of thickened eeebrow folds, widespread lesions, and positive slit-skin smears confirmed a diagnosis of LL with multibacillary disease.

Although our case hat no known medical illness such as diabetes or an immunocompromised state, working as a farmer in a rural area increases the risk of acquiring infections such as leptrspirosis, melioidosis, parasitic diseases and leprosy. Recent studies reported *M. leprae* DNA in soil from the homes of patients with leprosy and areas with infected animals, suggesting that soil may act as a temporary reservoir. This suggests a potential occupational exposure of our patientto *M. teprae* due lo frequent contact with soil.^9^

Leprosy is often mistaken for tinea versicolor, a superficial fungal infecticn caused by MaUfweia species, which presents as hypopigmented or pink plaques with fine scaling, predominantly on seborrheic areas such as the neck, chest, back, and abdomen, without sensory involvement. Diagnosis is clinical, supported by a positive potassium hydroxide (KOH) preparation, which is negative in leprosy cases.^10^ Vitiligo, an autoimmune disorder causing chalky white, symmetric patches of depigmentation, is another important differential diagnosis. Unlike leprosy, vitiligo is often associated with a family history of autoimmune diseases such as thyroiditis or alopecia areata.^10^ Pityriasis alba, commonly seen in children and young adults, manifests as ill-defined hypopigmented patches on the face, neck, and forearms and is typically self-limiting, often linked to atopic dermatitis.^10^

In contrast, PIH results from injury or inflammation and typically manifests as hyper- or hypopigmented patches without sensory loss. Inflammatory mediators such as prostaglandins, leukotrienes, and thromboxanes stimulate melanocyte hypertrophy and melanin overproduction.^10^ MF, a T-cell non-Hodgkin lymphoma, may also manifest skin lesions. Its hypopigmented variant, though uncommon, typically affects younger individuals and appears as circular or irregular hypopigmented patches or thin plaques with fine scaling, often asymptomatic or mildly pruritic. Unlike leprosy, MF diagnosis requires immunohistochemical confirmation.^11^

Accurate diagnosis depends on comprehensive history-taking, meticulous physical examination, and appropriate diagnostic testing. The slit-skin smear remains a key investigation, demonstrating 100% specificity and approximately 50% sensitivity.^12^ Optimal sampling sites include active or anesthetic lesions, earlobes, and the posterior shoulder. Detection of AFB is performed using modified Ziehl-Neelsen staining, with calculation of the Ridley-Jopling bacterial index.^12^

The final diagnosis in this case was multibacillary lepromatous leprosy, established based on clinical findings and confirmatory investigations. For therapeutic purposes, leprosy is classified as either paucibacillary (PB) or multibacillary (MB).^3^ PB leprosy is defined by one to five skin lesions with negative slit-skin smears, whereas MB leprosy is characterised by more than five skin lesions, nerve involvement (even with few or no lesions), or a positive slit-skin smear for bacilli.^3^

WHO recommends multidrug therapy for leprosy, with treatment duration determined by disease classification: six months for PB leprosy and twelve months for MB leprosy. In this case, he received multidrug therapy for twelve months, resulting in marked clinical improvement.

Contact tracing and health surveillance are essential to prevent community transmission. Early diagnosis and prompt treatment reduce the risk of complications, including nerve abscesses, neuritis, and peripheral neuropathies.^13^ Active case finding through community-based surveys and screening programmes is crucial for identifying new cases and initiating therapy before irreversible nerve damage occurs. Effective leprosy control requires a multifaceted approach that includes early detection, prompt multidrug treatment, contact tracing and disability prevention.^3^

## Conclusion

Leprosy should be suspected in patients with hypopigmented lesions accompanied by sensory loss, thickened peripheral nerves, or systemic manifestations, particularly in endemic regions. Differentiating leprosy from other causes of hypopigmented skin lesions is essential for timely, appropriate management and disability prevention. A structured approach that includes detailed history-taking, thorough physical examination, and targeted investigations such as KOH preparation or slit-skin smear is critical.
